# Supplementary material for: Structural insight into Tn3 family transposition mechanism
Source: Nat Commun. 2022 Oct 18;13:6155. doi: 10.1038/s41467-022-33871-z (PMC9579193; doi:10.1038/s41467-022-33871-z)
Supplement: Supplementary file 1 — Supplementary Information [file 41467_2022_33871_MOESM1_ESM.pdf]

Supplementary information

Structural insight into Tn3 family transposition mechanism

Alexander V. Shkumatov[1,2†], Nicolas Aryanpour[3], Cédric A. Oger[3], Gérôme Goossens[3$], Bernard F. Hallet[3*], and Rousland G. Efremov[1,2*]

[1] Center for Structural Biology, Vlaams Instituut voor Biotechnologie; Brussels, Belgium.

[2] Structural Biology Brussels, Department of Bioengineering Sciences, Vrije Universiteit Brussel; Brussels, Belgium.

[3]Louvain Institue of Biomolecular Science and Technology, Université Catholique de Louvain (UCLouvain); Croix du Sud 4/5, 1348 Louvain-la-Neuve, Belgium.

[*] Corresponding authors email address: bernard.hallet@uclouvain.be (BFH), rouslan.efremov@vub.be (RGE)

Present addresses: †Confo Therapeutics, Brussels, Belgium; [$]Thermo Fisher Scientific, Seneffe, Belgium

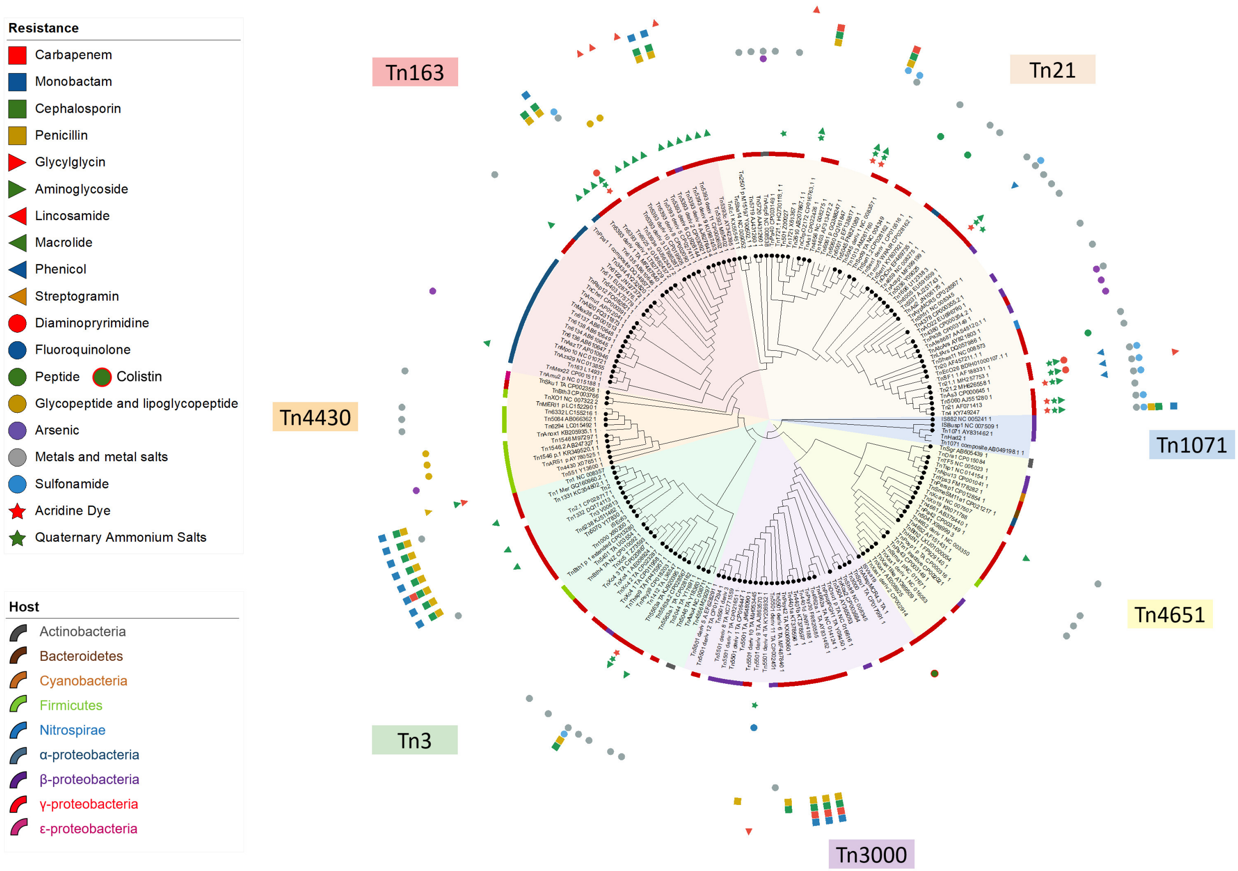

Supplementary Fig. 1 Antibiotic resistance in Tn3 family. A phylogenetic tree grouping 190 transposons of the Tn3 family shows the prevalence of antibiotic resistance genes within the family. The transposon subgroups are shaded in different colors. The different classes of antibiotics and therapeutic compounds against which the corresponding passenger genes confer resistance are indicated by specific symbols. The major groups of bacteria in which the transposons were initially identified are also shown. Evidence of transposition is indicated by black circles.

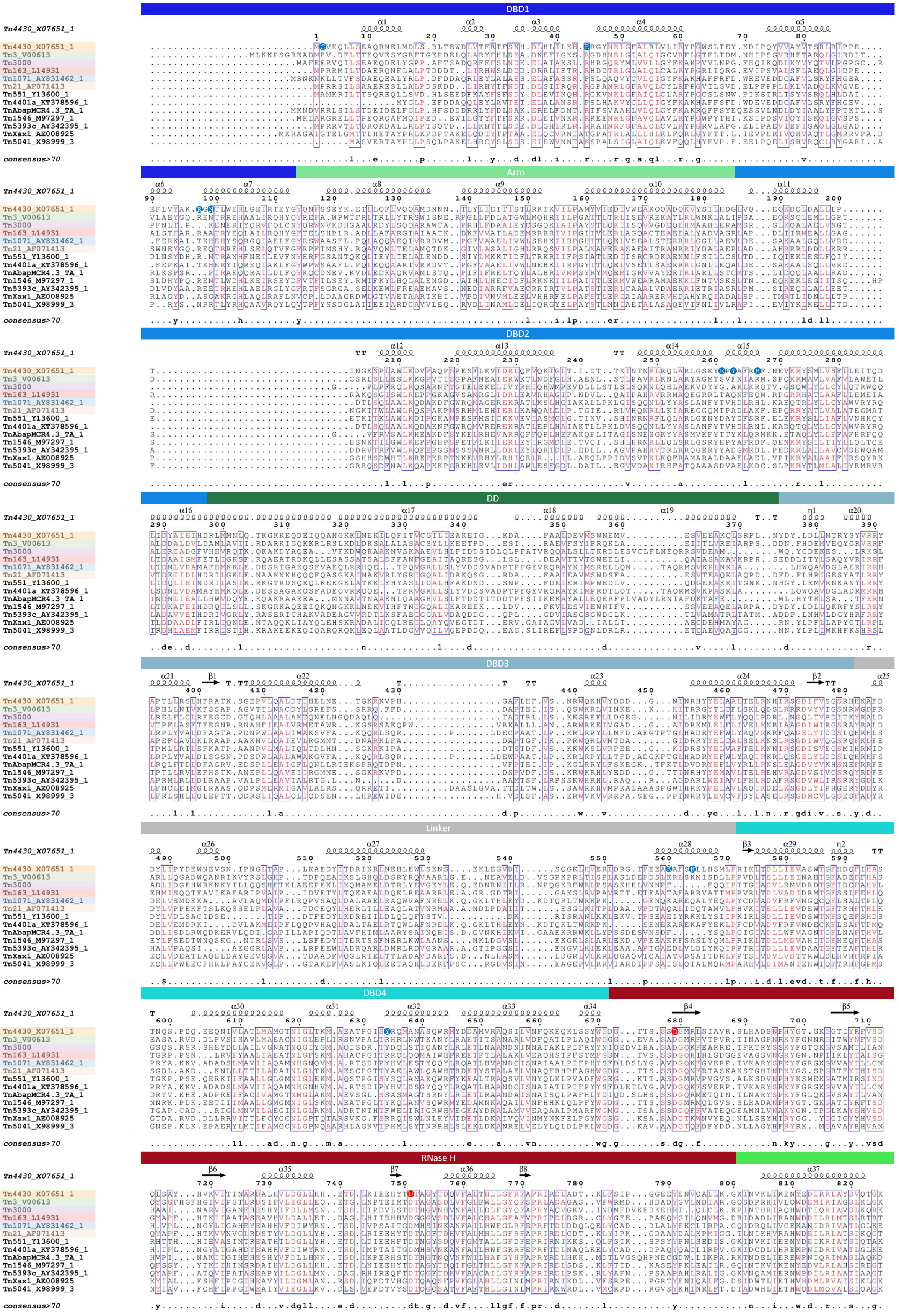
DBD1
Arm
DBD2
DD
DBD3
Linker
DBD4
RNase H
Tn4430_X07651_1
Tn3_V00613
Tn3000
Tn163_L14931
Tn1071_AY831462_1
Tn21_AF071413
Tn551_Y13600_1
Tn4401a_KT378596_1
TnAbapMCR4.3_TA_1
Tn1546_M97297_1
Tn5393c_AY342395_1
TnXax1_AE008925
Tn5041_X98999_3
consensus>70

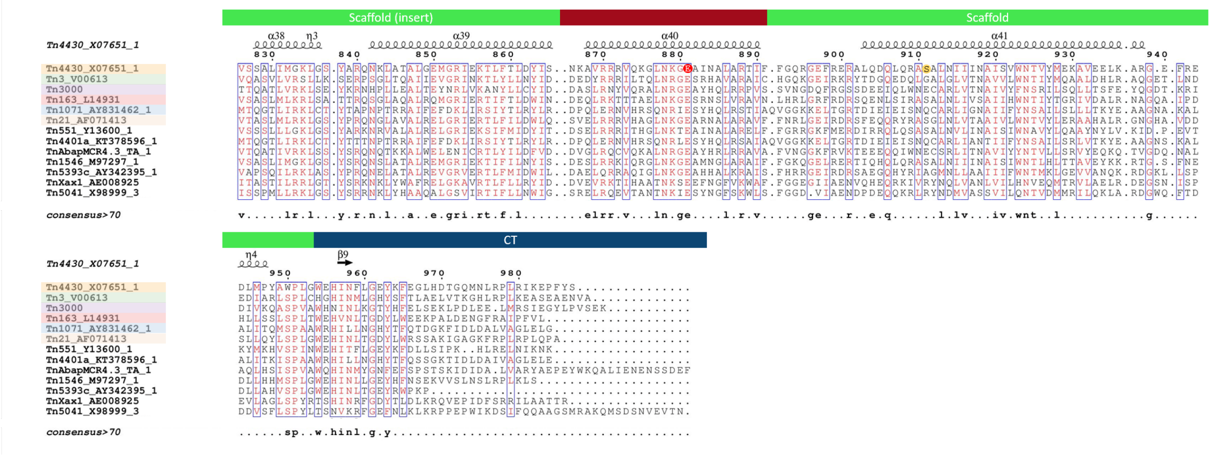

Supplementary Fig. 2 Alignment of TnpA from Tn3 family. Sequences of 157 TnpA genes from the Tn3 family covering complete phylogenetic tree from **Supplementary Fig. 1** were aligned with Clustal Omega and 13 TnpA sequences representative of the subfamily archetypes (coloured as in **Supplementary Fig. 1**) and resistance gene carriers are shown. The 70% consensus from the 157 Tn is displayed at the bottom of the sequences. The secondary structure of Tn4430 TnpA is shown schematically above the sequences and coloured with the corresponding domain colour (**Fig. 1c**). Catalytic residues are marked with red circles and residues forming sequence-specific interactions with DNA marked with blue circles.

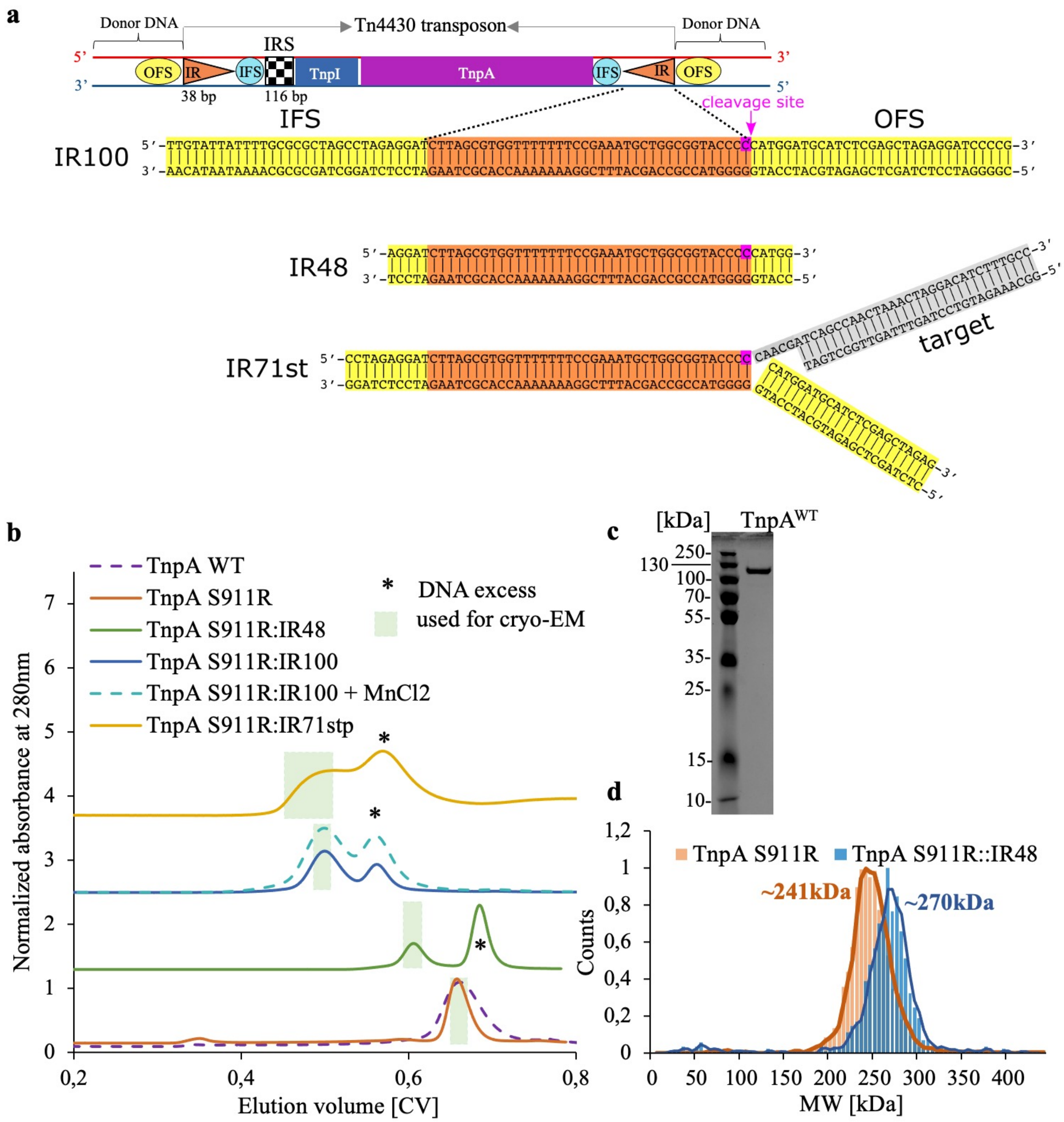

Supplementary Fig. 3 DNA substrates, purification, and characterization of TnpA. **a**, Structure of transposon and DNA substrates used for structure determination IFS: inner flanking sequence; OFS: outer flanking segment. **b**, Size exclusion chromatography profiles of TnpA with and without DNA substrates. The green rectangles correspond to the fractions used for cryo-EM. **c**, SDS-PAGE of purified TnpA (n>10). **d**, Mass photometry measurements indicate homogeneous TnpA populations in the apo state and in complex with IR100 substrate.

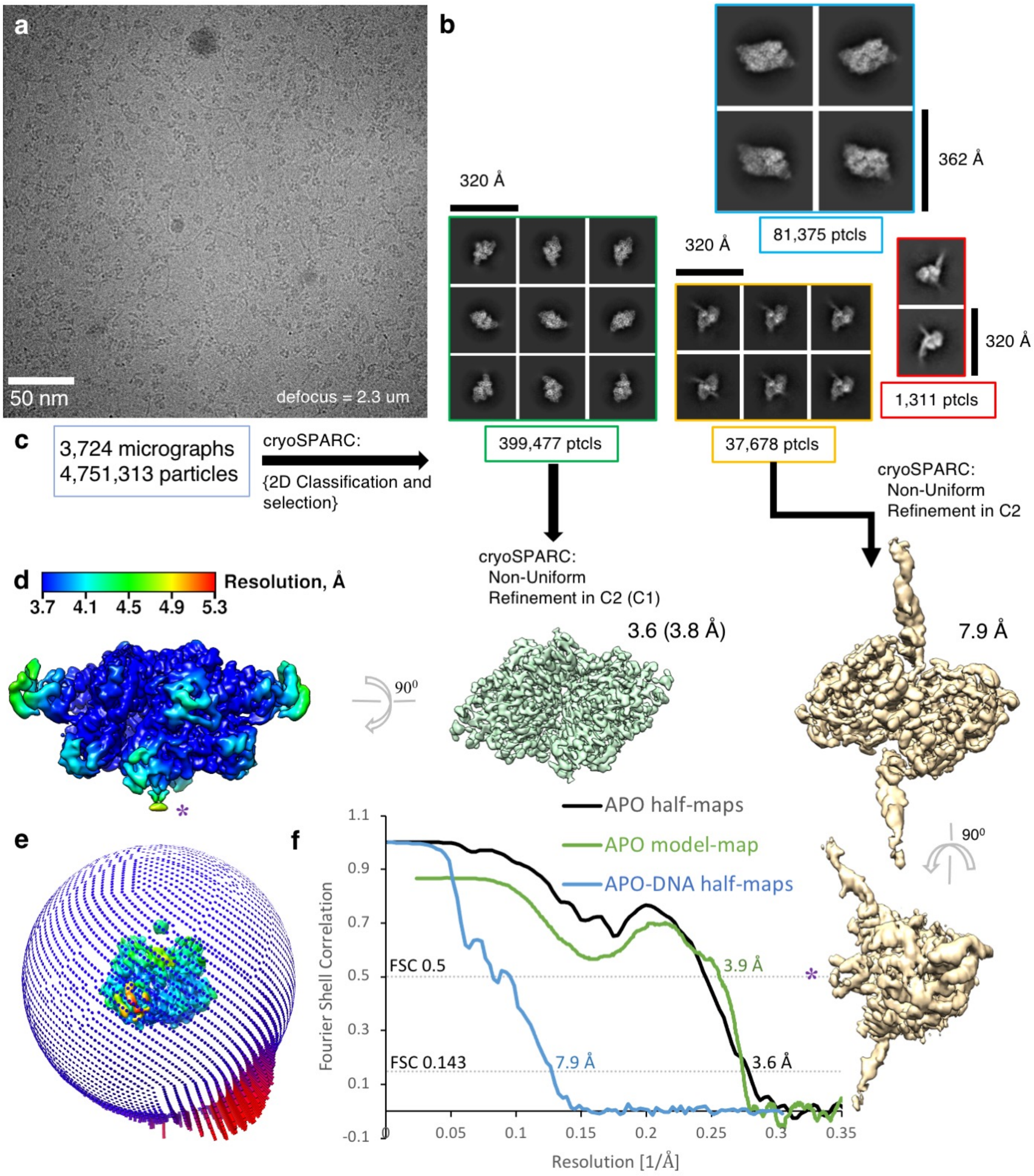

Supplementary Fig. 4 Cryo-EM reconstruction of TnpA^{WT} in the presence of IR100. a, Raw cryo-EM image. **b,** 2D class averages revealed several particle populations. **c,** Schematic representation of 3D reconstruction steps. Two reconstructions were determined from the dataset: 3.6 Å reconstruction of TnpA^{WT} apo state and 7.9 Å resolution TnpA^{WT}-IR100 complex. **d,** Local resolution of TnpA^{WT} reconstruction. **e,** Distribution of particle orientations. **f,** Fourier Shell Correlation (FSC) plots for half-maps, and between the model and cryo-EM map.

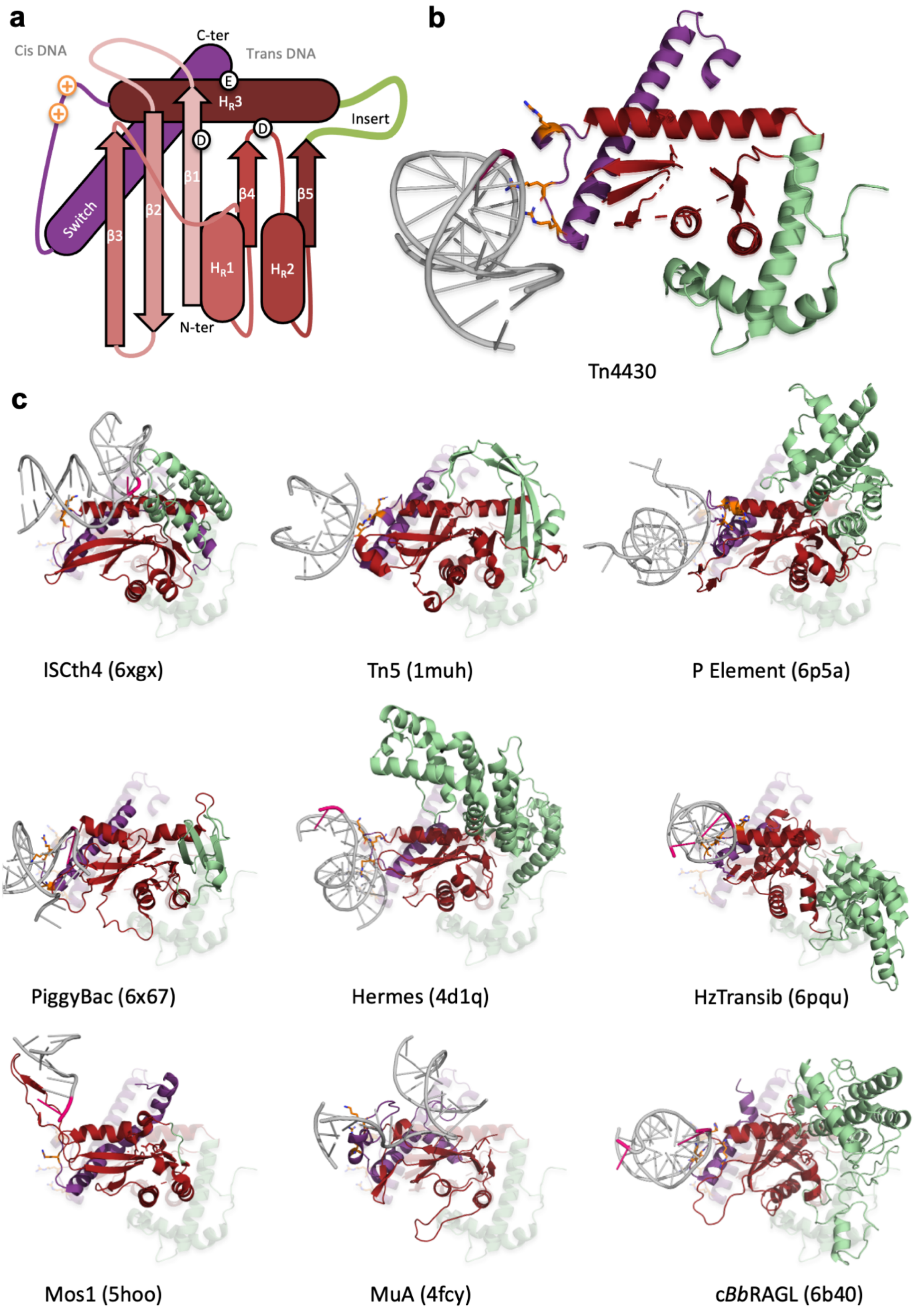
a
Cis DNA
C-ter
Trans DNA
E
H_R3
Insert
D
D
β1
β4
β5
β2
β3
Switch
H_R1
H_R2
N-ter
b
Tn4430
c
ISCth4 (6xgx)
Tn5 (1muh)
P Element (6p5a)
PiggyBac (6x67)
Hermes (4d1q)
HzTransib (6pqu)
Mos1 (5hoo)
MuA (4fcy)
cBbRAGL (6b40)

Supplementary Fig. 5 Switch helix and loop are conserved structural elements among transposases. a, Schematic figure showing fold of the RNH domain, the relative position of switch helix, switch loop, and insertion domain. The color coding of structural elements is applied to structures displayed in panels **b** and **c**. **b**, The structure of the RNH, the switch helix, and the insert domain in $TnpA^{S911R}$-IR100. **c**, The same functional module of other transposases in paired-end complex conformation is overlaid on that of TnpA shown as a transparent structure in the background. The structure of the insert domain of TnpA (light green) is different from the other transposases with known structure, whereas switch helix in extended form is found in all known transposases in a conformation similar to that of TnpA IR-bound complexes. The switch loop connecting H_R3 with switch helix carries positively charged residues that interact with transposon end in cis in all examined transposases except MuA, where it is directed at the target DNA and Mos1.

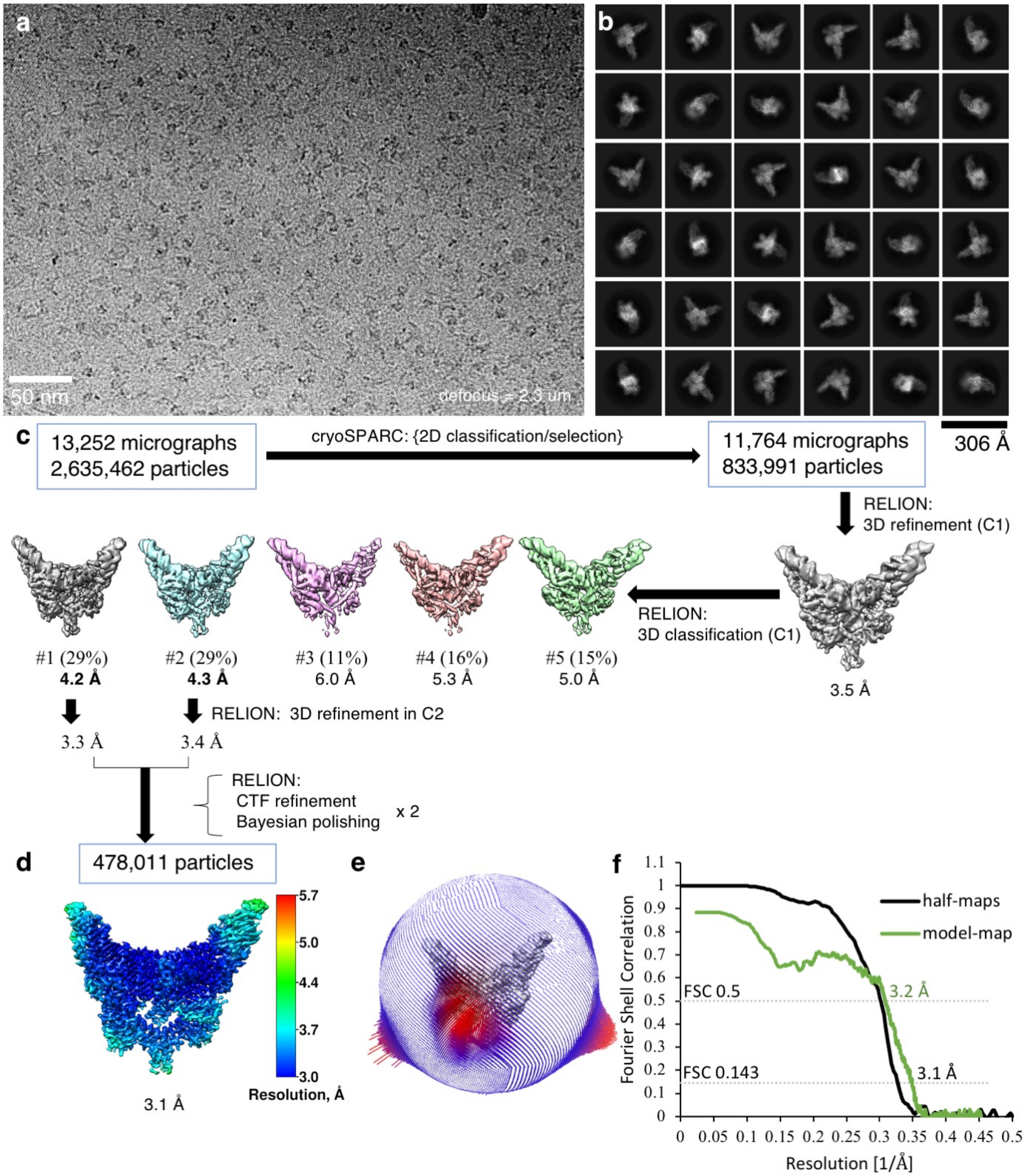

Supplementary Fig. 6 Cryo-EM reconstruction of the TnpA^{S911R}-IR48 complex. a, Raw cryo-EM image. **b,** 2D class averages. **c,** Schematic representation of 3D reconstruction steps. **d,** Local resolution of the reconstruction. **e,** Distribution of particle orientations. **f,** FSC plots for half-maps, and between the model and cryo-EM map.

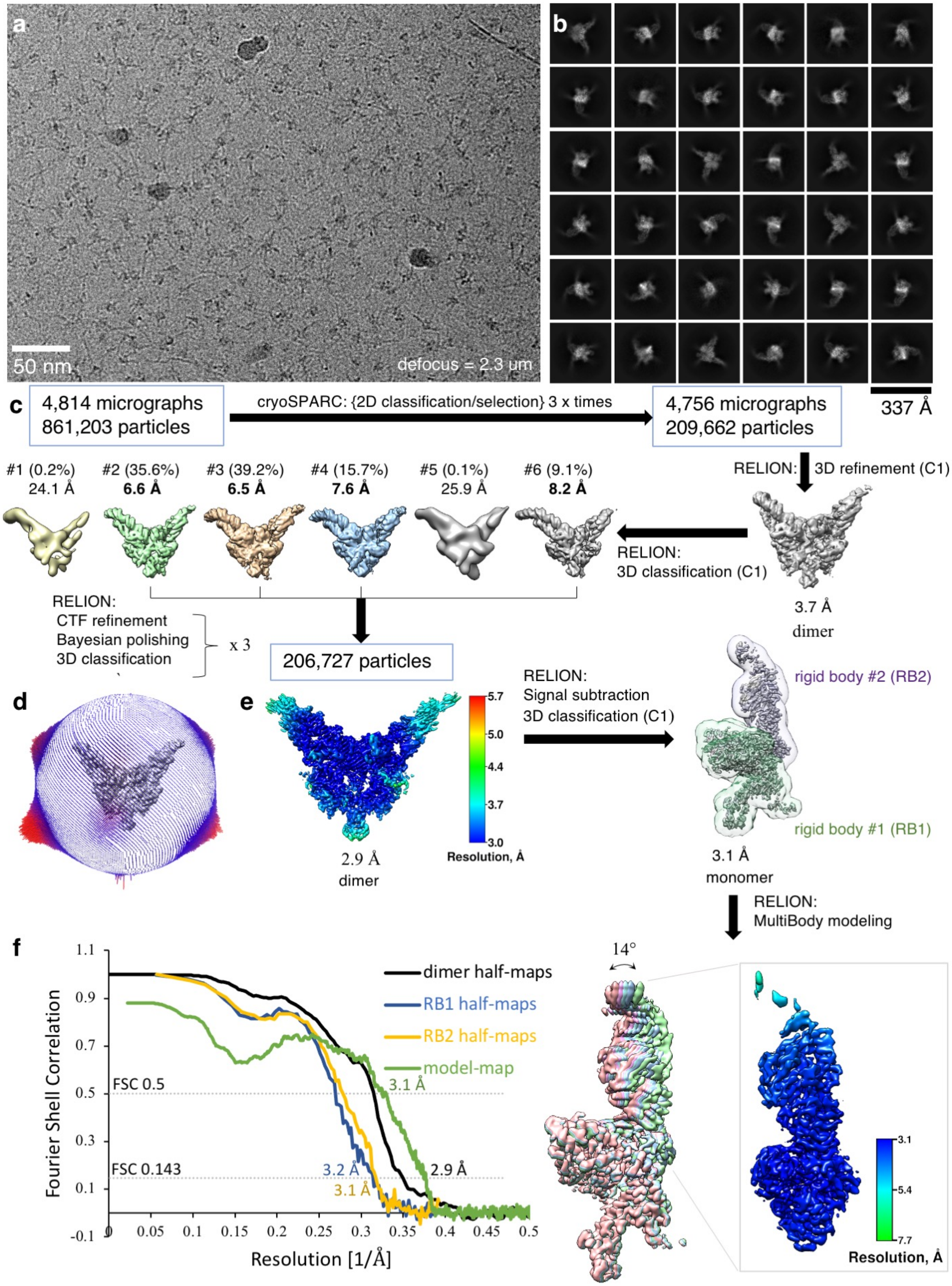

Supplementary Fig. 7 Cryo-EM reconstruction of the TnpA^{S911R}-IR100 complex. a, Raw

cryo-EM image. **b,** 2D class averages. **c,** Schematic representation of the 3D reconstruction steps and properties of the resulting reconstruction. The reconstruction of N-terminal DBD1 was improved by multi-body refinement. **d,** Distribution of particle orientations. **e,** Local resolution of the reconstruction. **f,** FSC plots for half-maps, and between the model and cryo-EM map.

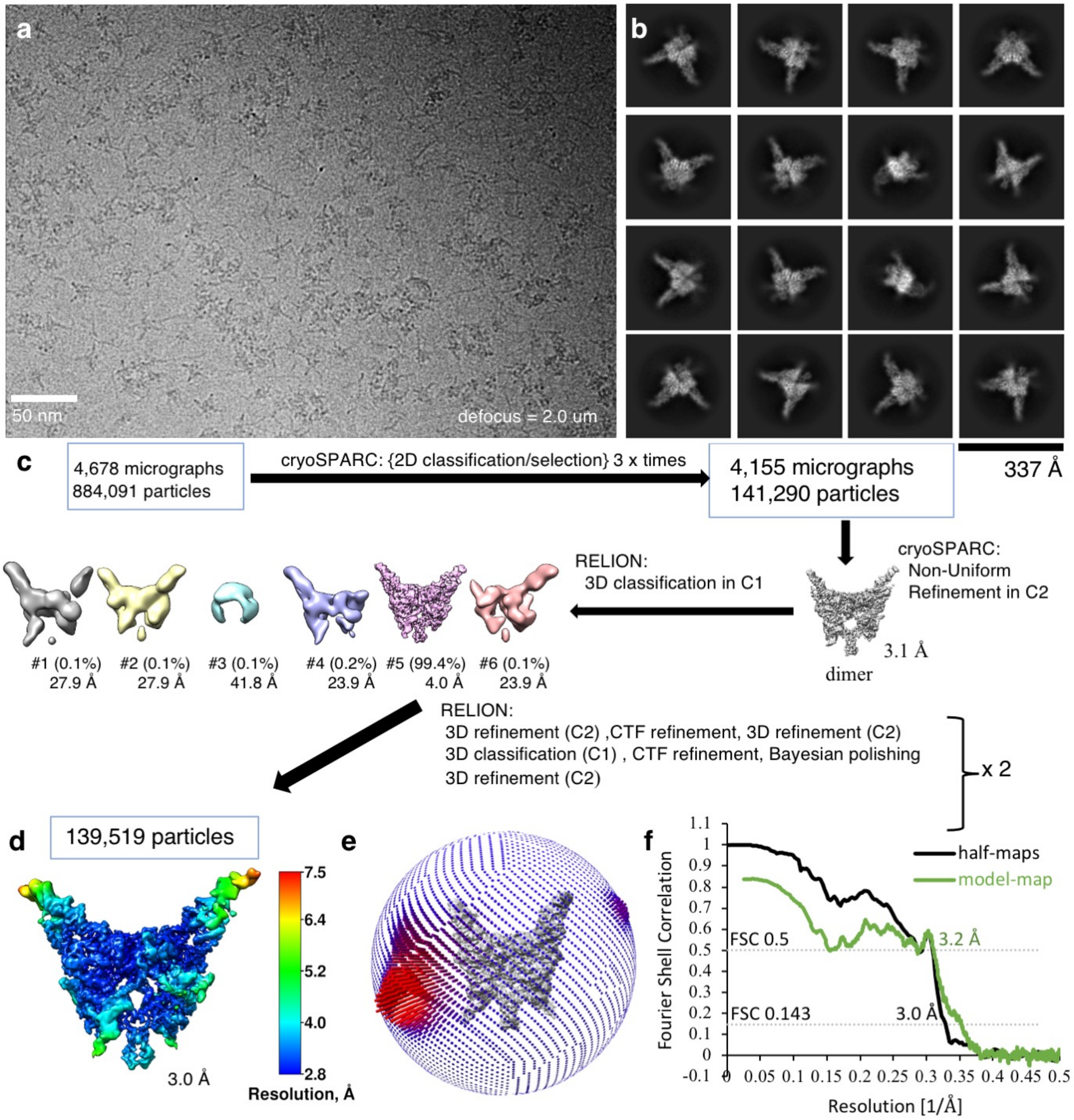

Supplementary Fig. 8 Cryo-EM reconstruction of the TnpA^{S911R}-IR71st complex. a, Raw cryo-EM image. **b,** 2D class averages. **c,** Schematic representation of 3D reconstruction steps, **d,** Local resolution of the reconstruction. **e,** Distribution of particle orientations. **f,** FSC plots for half-maps, and between the model and cryo-EM map.

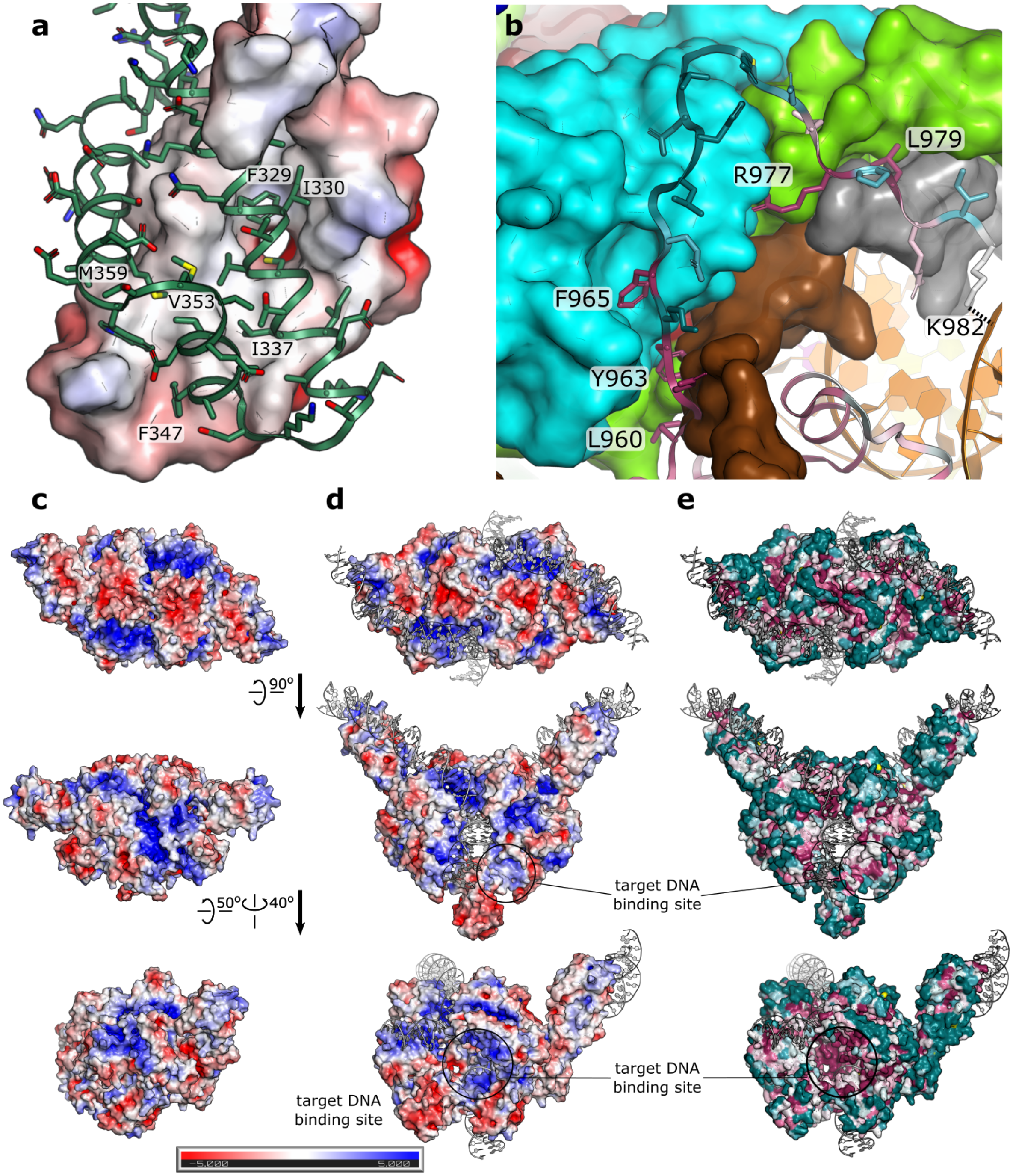

Supplementary Fig. 9 Dimerization interfaces and surface properties of TnpA. a, Interaction between DD domains involves nearly exclusively hydrophobic interactions. Surface electrostatic potential is shown for the DD of the second protomer. **b,** Dimerization interactions mediated by the C-terminus. Residues are colored based on the extent of conservation, as calculated by CONSURF[66]. Conservation increases from deep teal to white to purple. The surface of the adjacent protomer is colored by domains color-coded as shown in **Fig. 1d**. **c, d** Surface electrostatics for the apo state (**c**), and for $TnpA^{S911R}$-IR100 (**d**). **e**, Conservation of surface residues is shown for $TnpA^{S911R}$-IR100 as calculated by CONSURF. Protein orientation is same

as in panel **d**. Circles indicate surfaces with positive potential and high conservation suggesting putative position for target DNA binding.

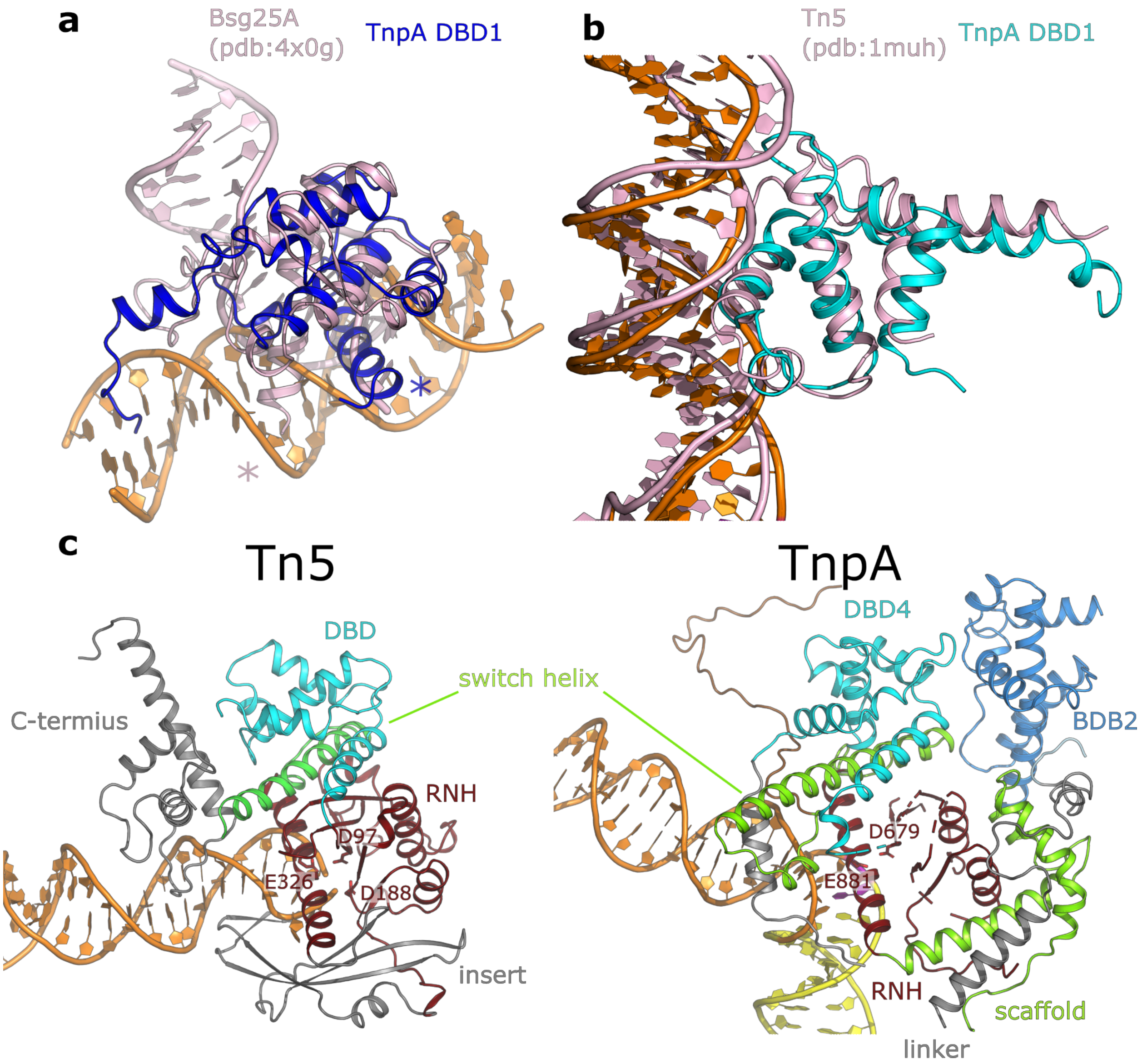

Supplementary Fig. 10 Structural homology of TnpA. a, DBD1 displays fold similarity to the BEN domain. Structural overlay is shown with Bsg25A from *Drosophila melanogaster*[37]; difference in orientation of bound DNA is likely due to difference in the position of DNA-reading helix marked with asterisks. **b,** Structural alignment of Tn5 (PDB identifier 1muh) cis-DNA binding domain to DBD4 of TnpA shows a similar fold and mode of DNA binding. **c**, Similarities, and differences in the architecture of Tn5 and TnpA. In Tn5, elements that structurally align with TnpA are color-coded as in **Fig. 1d**, whereas other domains, including insertion into the RNase H-like domain, are shown in grey. TnpA domains are color-coded as in **Fig. 1d**. Residues of the catalytic DDE tirade are indicated for both proteins. The orientation of the RNase H domains is similar in both proteins. For clarity, TnpA domains remote from the homologous core are not shown.

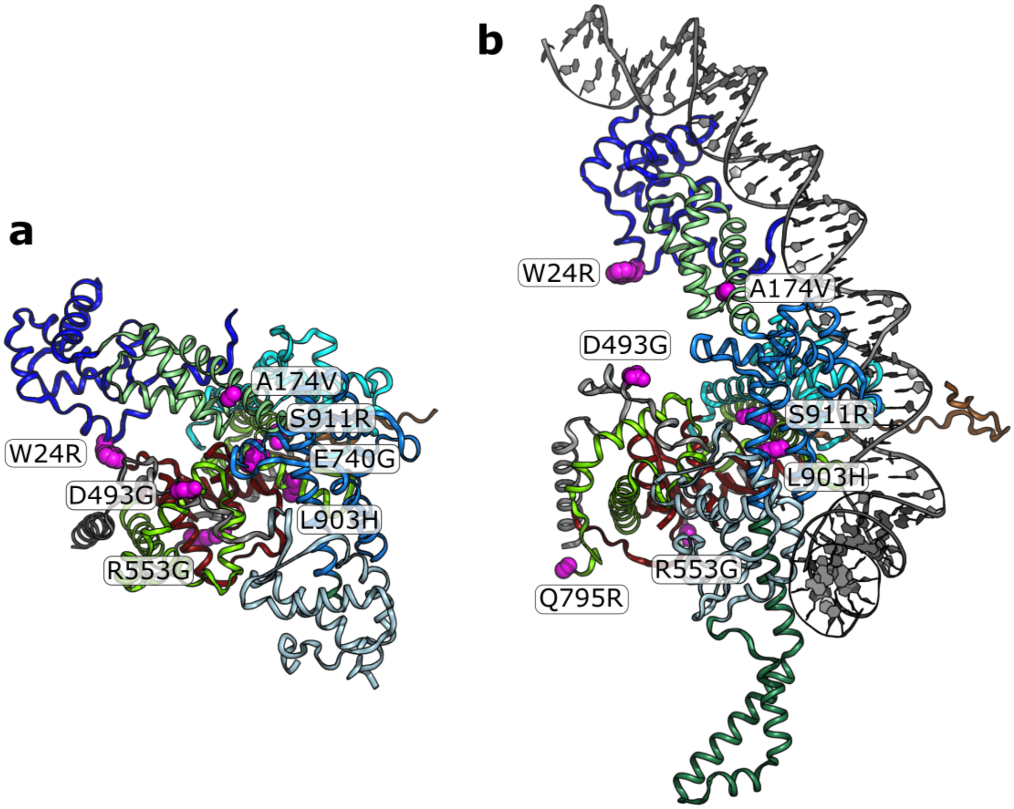

Supplementary Fig. 11 Distribution of hyperactivating mutations. Positions of hyperactive, immunity impairing mutations are shown as pink-sphere representations of the corresponding side chains. **a**, TnpA^{WT} apo conformation and **b,** TnpA^{S911R}-IR100 complex. The mutations are located at the interfaces between domains disrupted in the IR-bound conformation. All the mutations are found far from the protein-protein interface in the assembled dimer, therefore only monomers are shown for clarity.

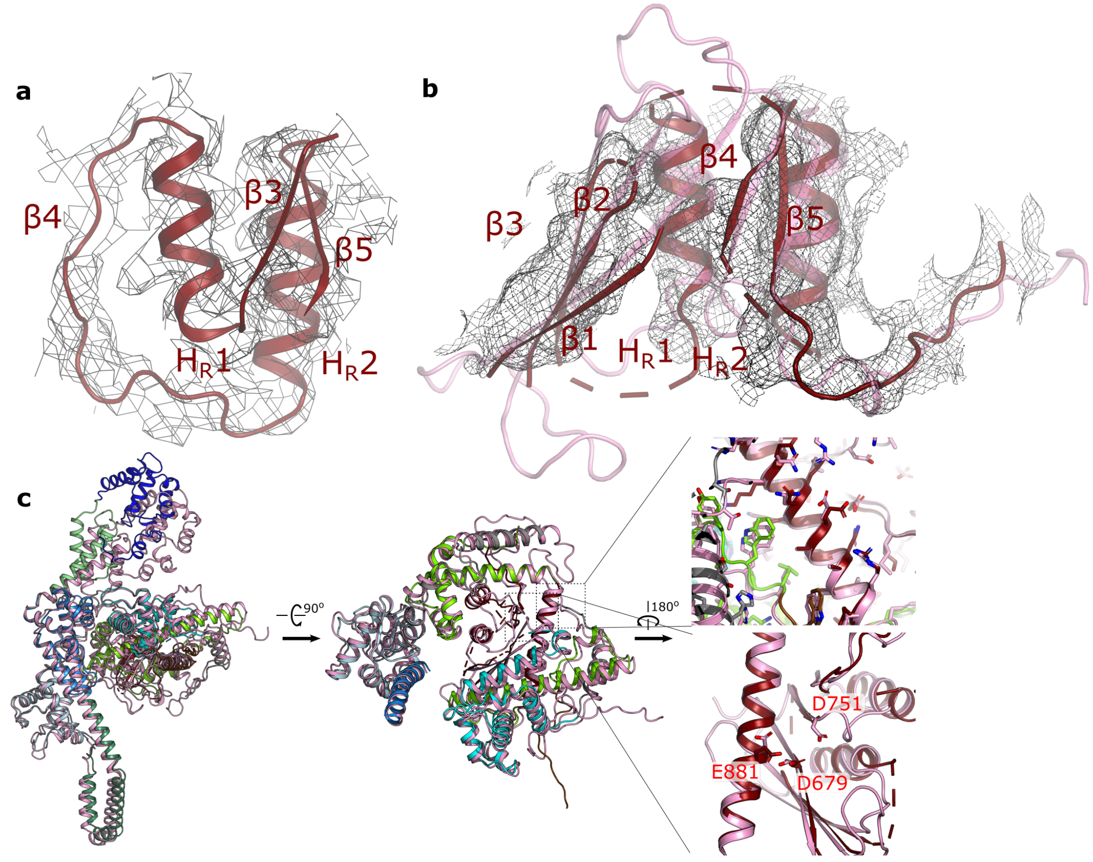

Supplementary Fig. 12 Modelling of the RNH domain and AlphaFold2 model of TnpA. a-b, Models of the RNH domain in apo (**a**) and TnpA^{S911R}-IR100 (**b**) states are shown (dark red) together with their cryo-EM density maps. In (**b**), the cryo-EM model is overlayed with predicted AlphaFold2 model of the RNH domain (light pink) showing that densities for the parts of the β-strands β1, β2 and β3 and connecting loops are missing. **c,** Overlay of TnpA model (colored by domains as in **Fig. 1d**) built into experimental map without the use of AphaFold2, except for RNH domain (bottom right panel), with the AplhaFold2 model (light pink). AlphaFold2 prediction of TnpA accurately matched the IR-bound conformation, albeit deviating at the position of DBD1. The AlphaFold2 model had a high predictive power and accurately traced polypeptides through density regions that were too poor for *ab initio* modeling. This included the 5-stranded β-sheet of the RNH domain. In the AphaFold2 model the catalytic triad D967, D751, and E881 is appropriately assembled to form an active site.

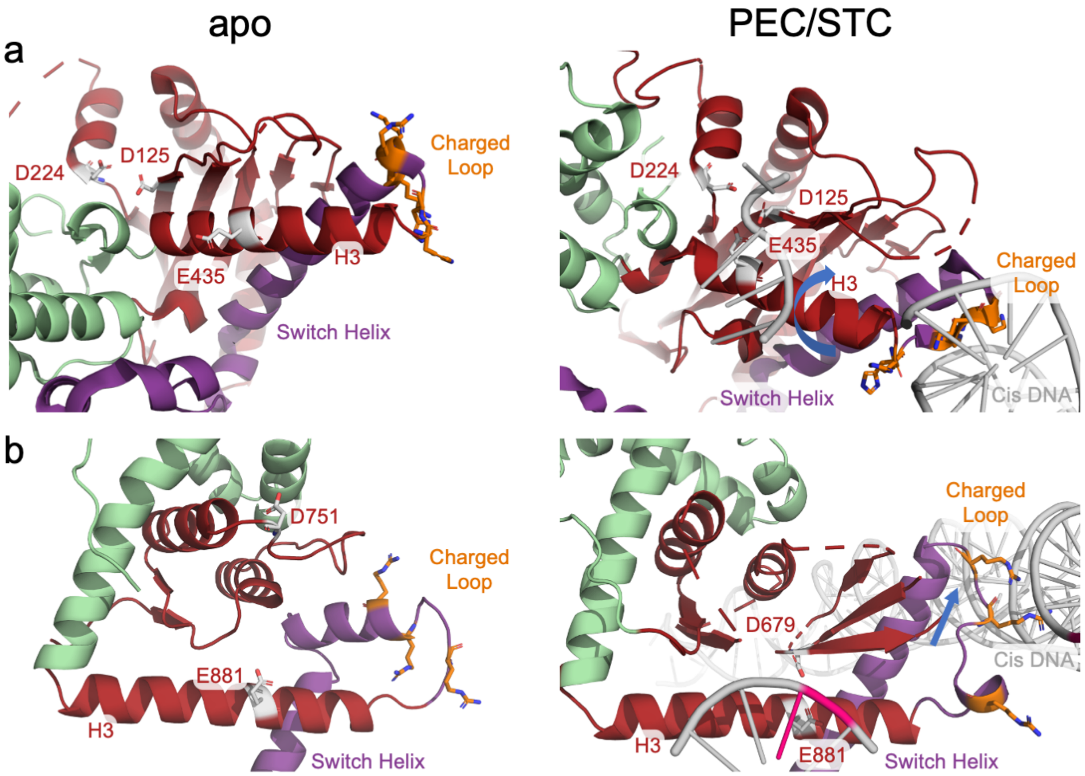

Supplementary Figure 13 The conformational changes in the active sites of Transib and TnpA. Active sites of Transib[32] (**a**) and TnpA (**b**) are shown in apo and transposon end bound conformation: strand transfer complex (STC) for Transib and IR-bound for TnpA. The RNH domain is shown in bordeaux, insertion in light green, switch helix in violet, and switch loop in orange. In DNA-bound conformation, cis DNA is shown as well. Conformational changes associated with switch loop movements: rotation of H_R3 in Transib and refolding of switch helix in TnpA are indicated by arrows.

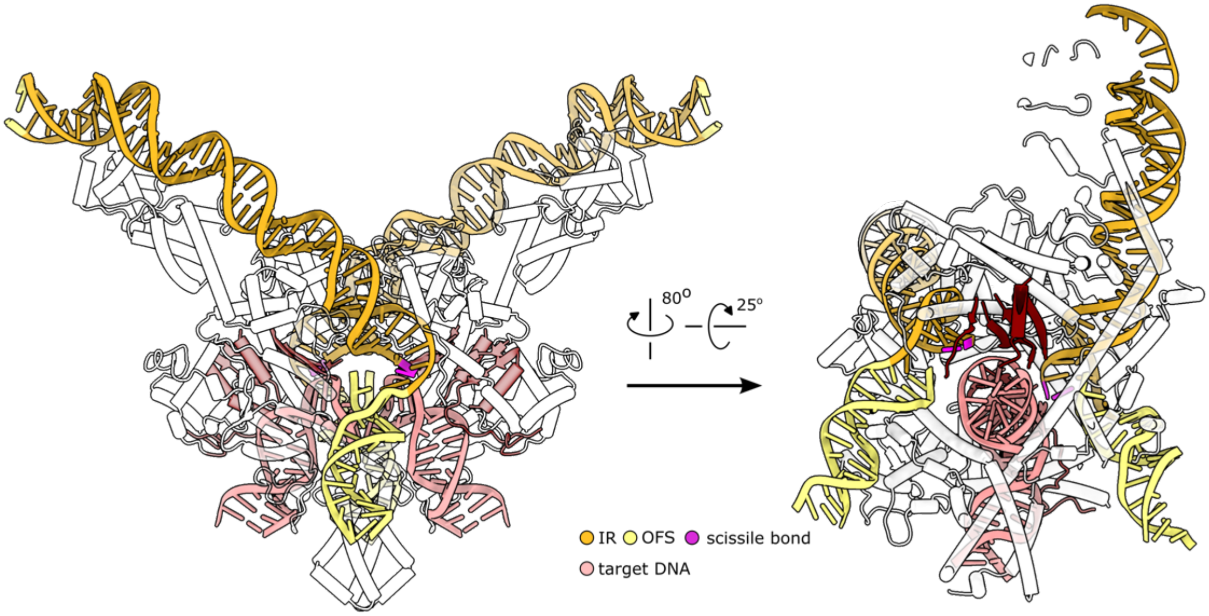

Supplementary Fig. 14 Putative model of target DNA in strand transfer complex. The structure of TnpA^{S911R}-IR71st complex is shown with a putative model of target DNA. The front part of the structure in the right panel is slabbed to better show the fit of the target DNA in the opening between the protomers. The protein is shown in cartoon representation as transparent grey with the RNH domain shown in bordeaux. To build the model, double helical DNA fragments were placed into the low-resolution density assigned to the target DNA branch (**Fig. 2d**) and a double-stranded fragment was placed in the opening between the TnpA protomers and the DD by aligning phosphates close to the scissile OH groups of cleaved transposon ends. The fragments were connected into a bent DNA, the model was minimized to eliminate steric clashes, and 2-fold symmetry was applied. Despite its very putative nature, the model of target DNA allows us to conclude that (1) a double-stranded DNA can be placed into the opening without major clashes, (2) number of nucleotides between putative strand transfer sites is equal to 5 as expected, and (3) the outer flanking segments of the donor DNA do not clash with target DNA.

Supplementary Table 1 Statistics of cryo-EM data collection and model refinement.

	**TnpA^{WT}**	**TnpA^{S911R} IR48**	**TnpA^{S911R} IR100**	**TnpA^{S911R} IR71st**
**Metal**	none	none	5mM $MnCl_2$	none
**Database accession codes**				
EMDB	EMD-1391	EMD-13908	EMD-13906	EMD-13909
RCSB	PDB-7QD8	PDB-7QD5	PDB-7QD4	PDB-7QD6
**Data collection**				
Microscope	FEI Titan Krios	JEOL CRYOARM300		
Energy filter / slit width [eV]	GIF Quantum energy filter (Gatan) / 20	In-column Omega energy filter / 20		
Detector	K2 summit (Gatan)	K3 Direct Detection Camera (Gatan)		
Nominal magnification	130 000	60 000		
Accelerating voltage [kV]	300	300		
Calibrated pixel size [Å]	1.067	0.764	0.766	0.760
Defocus range [µm]	0.6-5.3	0.6-4.7	1-6	0.4-3.2
Frames per movie	40	59	59	60
Exposure time, *s*	10	2.985	2.985	3.036
Total electron dose [e^-/Å^2]	50	62.5	62.5	63.6
Automation software	EPU v1.11	SerialEM v3.0.8		
Total movies used	3,724	11,764	4,756	4,155
**Reconstruction**				
Final particles [no.]	399,477	478,011	206,727	139,519
Box size [px]	300	440	440	440
Imposed symmetry	C2	C2	C2	C2
Sharpening B-factor, RELION [Å^2]	-177	-100	-60	-65
Resolution, RELION [Å] (FSC=0.143 / 0.5)	3.6 / 4.1	3.1 / 3.3	2.9 / 3.2	3.0 / 3.5
Local resolution range [Å]	3.4-6.3	2.9-5.7	2.8-4.7	2.8-8.1
3D FSC sphericity / Global resolution [Å]	0.8 / 3.6	0.9 / 3.0	0.9 / 2.9	0.7 / 3.1
**Refinement**				
Map sharpening B-factor (Å2)	- 187.2	-99.6	-60.3	-59.8
Software for real-space refinement	Phenix 1.19.2	Phenix 1.19.2	Phenix 1.19.2	Phenix 1.19.2
Model resolution at FSC=0.5 (Å)	3.9	3.2	3.1	3.2
Model composition				
Nonhydrogen atoms	14088	18386	19124	19118
Protein residues	1730	1816	1816	1816
Nucleotides	0	180	216	216
Ligand	0	0	0	0
B factors (Å^2)				
Protein	46.6	12.4	28.8	86.38
Nucleotide	--	24.1	53.2	167.84
Ligand	--	--	--	--
R.m.s. deviations				
Bond lengths (Å)	0.004	0.005	0.004	0.005
Bond angles (°)	0.744	0.823	0.821	0.886
**Validation**				
MolProbity score	1.35	1.06	1.17	1.32
Clashscore	5.14	2.08	3.37	4.29
Poor rotamers (%)	0	0.13	0	0
Ramachandran plot				
Favored (%)	97.66	97.64	97.81	97.42
Allowed (%)	2.34	2.36	2.19	2.58
Disallowed (%)	0	0	0	0

Supplementary Table 2 Modeled proteins and DNA.

Model	Protein residues	DNA base pairs, 5′ strand
$TnpA^{WT}$	2-303, 376-440, 452-530, 547-670, 715-772, 797-966	NA
$TnpA^{S911R}$-IR48		-4 to 41
$TnpA^{S911R}$-IR100	2-530, 55-671, 678-684, 703-709, 718-723, 726-738, 746-750, 754-784, 795-982	-13 to 41
$TnpA^{S911R}$-IR71st		-13 to 41

Supplementary Table 3 Results of fold similarity search with DALI. Top matches are shown for the corresponding domains. Z score is relatively low for most of the domains and in most cases reflects the only similarity in the packing of the helices between the domains but not the actual connectivity. The domains which are topologically similar are highlighted in bold.

Domain	DALI results						
	**Molecule name**	**Z score**	**Rmsd, [Å]**	**Length alignment**	**N residues**	**% identity**	**PDB / chain ID**
**DBD1** (1-114)	Ben domain-containing protein 3	5.6	3.2	69	97	7	5jno/A
arm (115-185)	Polyribonucleotide nucleotidyltransferase	7.5	2.6	65	659	11	4nbq/A
DBD2 (186-298)	Actin	6.7	2.6	70	598	9	5ce3/B
DD (299-374)	Envelope glycoprotein gp160, gp41 chr region	5.7	6.0	71	77	10	5hfm/e
DBD3 (375-481)	Programmed cell death 6-interacting protein	4.3	3.6	72	697	7	2oev/A
DBD4 (572-671)	Transcription factor tfiib-like	5.1	2.7	74	207	4	3h4c/A
**RNH-SDF** (672-953)	Transposase for transposon tn5	5.5	4.4	150	460	13	4dm0/A

Supplementary Table 4 Properties of TnpA interfaces. Contacts analyzed by PISA program.

Interacting domains	apo		IR100		IR48		IR71st	
	surface [Å^2]	ΔG [kcal/ mol]	surface [Å^2]	ΔG [kcal/ mol]	surface [Å^2]	ΔG [kcal/ mol]	surface [Å^2]	ΔG [kcal/ mol]
Dimerization C-terminus	1590.3	-16.0	3015	-31.3	3041	-23.6	2963	-19.5
DD domains	NA*	NA	1607	-35.5	1649	-34.3	1756	-31.9
cis-DNA-TnpA	NA	NA	3337	-36.2	2729	-26.0	3387	-34.1
trans DNA-TnpA	NA	NA	855	-12.0	859	-8.9	743	-6.6

* No atomic resolution structure of the domain is available for the apo state.

Supplementary Table 5 DNA substrates used in this study.

Substrate name	Oligonucleotide name	5′ to 3′ sequence	MW, kDa
IR48	GG IR48-1 Full	AGGATCTTAGCGTGGTTTTTTTCCGAAATGCTGGCGGTACCCCCATGG	14.9
	GG IR48-2 Full	CCATGGGGGTACCGCCAGCATTTCGGAAAAAAACCACGCTAAGATCCT	14.8
IR100	EN100-2long	TTGTATTATTTTGCGCGCTAGCCTAGAGGATCTTAGCGTGGTTTTTTTCCGAAATGCTGGCGGTACCCCCATGGATGCATCTCGAGCTAGAGGATCCCCG	31
	EN100-2long	CGGGGATCCTCTAGCTCGAGATGCATCCATGGGGGTACCGCCAGCATTTCGGAAAAAAACCACGCTAAGATCCTCTAGGCTAGCGCGCAAAATAATACAA	31
IR71stp	NA IR71	CTCTAGCTCGAGATGCATCCATGGGGGTACCGCCAGCATTTCGGAAAAAAACCACGCTAAGATCCTCTAGG	21.9
	NA IR71short	CATGGATGCATCTCGAGCTAGAG	7.2
	NLC710	GGCAAAGATGTCCTAGTTTAGTTGGCTGAT	9.4
	NA IR71-NLC707	CCTAGAGGATCTTAGCGTGGTTTTTTTCCGAAATGCTGGCGGTACCCCCAACGATCAGCCAACTAAACTAGGACATCTTTGCC	25.6
