# Supplementary material for: Structural insight into Tn3 family transposition mechanism
Source: Nat Commun. 2022 Oct 18;13:6155. doi: 10.1038/s41467-022-33871-z (PMC9579193; doi:10.1038/s41467-022-33871-z)
Supplement: Supplementary file 3 — Description of Additional Supplementary Files [file 41467_2022_33871_MOESM3_ESM.pdf]

Description of Additional Supplementary Files

File Name: Supplementary Movie 1
Description: Architecture of TnpA in the apo and PEC conformations. The video shows the arrangement of subunits in the TnpA dimer and the conformation of the complex in both the apo and PEC conformations. The presence of a large opening that can accommodate the target DNA between the protomers in PEC is evident. The domains are colored as shown in Fig. 1d.

File Name: Supplementary Movie 2
Description: Morphs between conformations of DNA outer flanking sequences (OFS) for DNA substrates IR48, IR100, and IR71st.

File Name: Supplementary Movie 3
Description: Conformational transition from the apo to TnpAS911R-IR100 conformation. DBDs 1-3 move as rigid bodies upon PEC formation. The conformational transition opens DBD3 for OFS binding, rearranges DBD2 relative to DBD4, enables IR binding, and changes the fold of the switch helix HS, permitting refolding and assembly of the RNH domain. The morphs are shown only to visualize the differences in the conformations between two structures and should not be considered as the visualization of the real conformational transition place during transpososome assembly, which is expected to transit via an intermediate step with bound target DNA.
