# Supplementary material for: Structural insight into Tn3 family transposition mechanism
Source: Nat Commun. 2022 Oct 18;13:6155. doi: 10.1038/s41467-022-33871-z (PMC9579193; doi:10.1038/s41467-022-33871-z)
Supplement: Supplementary file 7 — Reporting Summary [file 41467_2022_33871_MOESM7_ESM.pdf]

nature portfolio

Corresponding author(s): Bernard F. Hallet, Rouslan G. Efremov

Last updated by author(s): 23/09/22

Reporting Summary

Nature Portfolio wishes to improve the reproducibility of the work that we publish. This form provides structure for consistency and transparency in reporting. For further information on Nature Portfolio policies, see our Editorial Policies and the Editorial Policy Checklist.

Statistics

For all statistical analyses, confirm that the following items are present in the figure legend, table legend, main text, or Methods section.

n/a	Confirmed	
☐	☒	The exact sample size (*n*) for each experimental group/condition, given as a discrete number and unit of measurement
☒	☐	A statement on whether measurements were taken from distinct samples or whether the same sample was measured repeatedly
☒	☐	The statistical test(s) used AND whether they are one- or two-sided<br>*Only common tests should be described solely by name; describe more complex techniques in the Methods section.*
☒	☐	A description of all covariates tested
☒	☐	A description of any assumptions or corrections, such as tests of normality and adjustment for multiple comparisons
☒	☐	A full description of the statistical parameters including central tendency (e.g. means) or other basic estimates (e.g. regression coefficient) AND variation (e.g. standard deviation) or associated estimates of uncertainty (e.g. confidence intervals)
☒	☐	For null hypothesis testing, the test statistic (e.g. F, t, r) with confidence intervals, effect sizes, degrees of freedom and P value noted<br>*Give P values as exact values whenever suitable.*
☒	☐	For Bayesian analysis, information on the choice of priors and Markov chain Monte Carlo settings
☒	☐	For hierarchical and complex designs, identification of the appropriate level for tests and full reporting of outcomes
☒	☐	Estimates of effect sizes (e.g. Cohen's *d*, Pearson's *r*), indicating how they were calculated

Our web collection on statistics for biologists contains articles on many of the points above.

Software and code

Policy information about availability of computer code

Data collection	Serial EM scripts used for single particle cryo-EM data collection on cryoARM300 electron microscope are available at https://github.com/BECM-VIB/SerialEM_scripts, SerialEM v3.0.8, EPU v1.11
Data analysis	RELION_IT, MotionCorr2, CTFFIND-4, cryoSPARC v3.1.0 , RELION v3.1, PHENIX v1.19.1, COOT v0.9.5, PHEINIX v1.19.2 , MolProbity v4.2

For manuscripts utilizing custom algorithms or software that are central to the research but not yet described in published literature, software must be made available to editors and reviewers. We strongly encourage code deposition in a community repository (e.g. GitHub). See the Nature Portfolio guidelines for submitting code & software for further information.

Data

Policy information about availability of data

All manuscripts must include a data availability statement. This statement should provide the following information, where applicable:

- Accession codes, unique identifiers, or web links for publicly available datasets
- A description of any restrictions on data availability
- For clinical datasets or third party data, please ensure that the statement adheres to our policy

Cryo-EM density maps and atomic models were deposited in the PDB and EMDB databases under the followingaccession codes: TnpAWT (PDB ID: 7QD8, EMD-13910), TnpAS911R-IR100 (PDB ID: 7QD4, EMD-13906), TnpAS911R-IR48 (PDB ID: 7QD5, EMD-13908), and TnpAS911R-IR71st (PDB ID: 7QD6, EMD-13909). The atomic models used in this study are available in the PDB database under accession code 4X0G, 1MUH, 6PQN, 6PR5, 6XGX, 6P5A, 6X67, 4D1Q, 6PQU, 5HOO, 4FCY, 6B40.

March 2021

Field-specific reporting

Please select the one below that is the best fit for your research. If you are not sure, read the appropriate sections before making your selection.

☒ Life sciences ☐ Behavioural & social sciences ☐ Ecological, evolutionary & environmental sciences

For a reference copy of the document with all sections, see nature.com/documents/nr-reporting-summary-flat.pdf

Life sciences study design

All studies must disclose on these points even when the disclosure is negative.

Sample size	The numbers of single particles used for calculating 3D reconstructions is provided in the Supplementary Table 1
Data exclusions	No data were excluded
Replication	Single particle cryo-EM data contain internal multiplicity and analyzed using statistical approaches to validate the results like independent processing of separate half of the datasets. For this reason, the collection of multiple datasets is not needed to validate the 3D reconstructions.
Randomization	Randomization was applied in the form of random particle selection for calculating independent 3D reconstruction for calculating gold-standard FSC curves used for regularization.
Blinding	Blinding was not used in 3D cryo-EM single particle analysis.

Reporting for specific materials, systems and methods

We require information from authors about some types of materials, experimental systems and methods used in many studies. Here, indicate whether each material, system or method listed is relevant to your study. If you are not sure if a list item applies to your research, read the appropriate section before selecting a response.

Materials & experimental systems

n/a	Involved in the study	
☒	☐	Antibodies
☒	☐	Eukaryotic cell lines
☒	☐	Palaeontology and archaeology
☒	☐	Animals and other organisms
☒	☐	Human research participants
☒	☐	Clinical data
☒	☐	Dual use research of concern

Methods

n/a	Involved in the study	
☒	☐	ChIP-seq
☒	☐	Flow cytometry
☒	☐	MRI-based neuroimaging
